# Reply: Paternal age and neonatal health: unraveling epigenetic pathways

**DOI:** 10.1093/hropen/hoaf053

**Published:** 2025-09-01

**Authors:** Wenxue Xiong, Xijia Tang, Lu Han, Li Ling

**Affiliations:** Department of Medical Statistics, School of Public Health, Sun Yat-sen University, Guangzhou, Guangdong, China; Department of Medical Statistics, School of Public Health, Sun Yat-sen University, Guangzhou, Guangdong, China; National Health Commission Key Laboratory of Male Reproduction and Genetics, Guangdong Provincial Reproductive Science Institute (Guangdong Provincial Fertility Hospital), Guangzhou, China; Department of Medical Statistics, School of Public Health, Sun Yat-sen University, Guangzhou, Guangdong, China; Clinical Research Design Division, Clinical Research Center, Sun Yat-sen Memorial Hospital, Sun Yat-sen University, Guangzhou, Guangdong, China

Dear Editor,

We would like to thank Drs Yuan and Zhao for their interest in our article (Xiong *et al.*, 2025) and their insights (Yuan and Zhao, 2025). However, first of all, we must clarify that we did not ignore the interaction effect of maternal and paternal age in our study. To investigate the interaction between maternal and paternal age, we first stratified the analyses by female age. After stratification by maternal age at the last menstrual period, increasing paternal age remained significantly associated with cesarean delivery and preterm birth (PTB), with similar trends across all stratums for maternal age except for mothers aged ≥35 years (please refer to Supplementary Figure S3 in Xiong *et al.* (2025)). In addition, we evaluated whether the combined effects of paternal and maternal age on cesarean delivery and PTB were more or less additive, which was considered to be most appropriate for assessing the public health importance of such interactions (Knol and VanderWeele, 2012). We found that the combined effects of advanced paternal and maternal age were less than additive joint effects (relative excess risk due to interaction < 0) (please see Table 3 in Xiong *et al.* (2025)). Furthermore, although our article (Xiong *et al.,* 2025) was focused on the association between paternal age and neonatal outcomes, we are interested in the long-term health effects of paternal age on the offspring. However, this was not possible for this study as the National Free Preconception Checkups Project (NFPCP) does not collect long-term data.

We agree that the biological mechanisms behind these associations deserve further investigation. However, it is worth noting that paternal age is both a ‘biological fact’ and a ‘social construct’ (Nybo Andersen and Urhoj, 2017). Previous studies have shown that advanced paternal age is associated with negative health behaviors (such as smoking, alcohol consumption, and obesity) and medical conditions (such as subfertility), which have also been linked to adverse perinatal outcomes (Nilsen *et al.*, 2013; DoPierala *et al.*, 2016; Brown, 2018). From a public health perspective, whether the association was driven by biological or selection factors, the impact warrants attention (Brown, 2018).
